# Plasma lipoprotein subfraction concentrations are associated with lipid metabolism and age-related macular degeneration[S]

**DOI:** 10.1194/jlr.M073684

**Published:** 2017-07-11

**Authors:** Chui Ming Gemmy Cheung, Alfred Gan, Qiao Fan, Miao Ling Chee, Rajendra S. Apte, Chiea Chuen Khor, Ian Yeo, Ranjana Mathur, Ching-Yu Cheng, Tien Yin Wong, E. Shyong Tai

**Affiliations:** Singapore Eye Research Institute,* Singapore National Eye Centre, Singapore; Department of Ophthalmology, Yong Loo Lin School of Medicine,† National University of Singapore, Singapore; Department of Medicine, Cardiovascular and Metabolic Disorders Programme,§§ National University of Singapore, Singapore; Centre for Quantitative Medicine,§ Duke-NUS Medical School, National University of Singapore, Singapore; Ophthalmology and Visual Sciences Program,*** Duke-NUS Medical School, National University of Singapore, Singapore; Ophthalmology and Visual Sciences,** Developmental Biology and Medicine, Washington University School of Medicine, St. Louis, MO; Genome Institute of Singapore,†† Singapore

**Keywords:** high density lipoprotein, genetics, cholesterylester transfer protein

## Abstract

Disturbance in lipid metabolism has been suggested as a major pathogenic factor for age-related macular degeneration (AMD). Conventional lipid measures have been inconsistently associated with AMD. Other factors that can alter lipid metabolism include lipoprotein phenotype and genetic mutations. We performed a case-control study to examine the association between lipoprotein profile and neovascular AMD (nAMD) and whether the cholesterylester transfer protein (*CETP*) D442G mutation modulates these associations. Patients with nAMD had significantly higher concentrations of HDL and IDL compared with controls. The increase in HDL particles in nAMD patients was driven by an excess of medium-sized particles. Concurrently, patients with nAMD also had lower Apo A-1, lower VLDL and chylomicron lipoprotein. Many of these associations showed a dose-dependent association between controls, early AMD cases, and nAMD cases. Adjustment for the presence of the D442G mutation at the *CETP* locus did not significantly alter the increased AMD risk associated with HDL particle concentration. AMD is associated with variation in many lipoprotein subclasses, including increased HDL and IDL particles and decreased Apo A-1, VLDL, and chylomicron particles. These data suggest widespread systemic disturbance in lipid metabolism in the pathogenesis of AMD, including possible alterations in lipoprotein carrier capacity.

Age-related macular degeneration (AMD) is one of the major causes of blindness worldwide (1, 2). Despite extensive research, the pathogenesis of AMD remains elusive and is likely multifactorial, involving genetic, lifestyle, and systemic factors (3–5). Current treatment in the form of anti-vascular endothelial growth factor therapy mainly addresses the specific angiogenic complications of neovascular AMD (nAMD). However, the response to these drugs varies among individuals, and some eyes are unresponsive to this therapy (6–10). Additional pathways other than angiogenesis may also play significant roles in the pathogenesis of AMD and provide potential alternative means of therapy.

In this regard, several pathways have been implicated in the pathogenesis of AMD, including chronic inflammation, atherosclerosis, and lipid dysregulation (3, 4, 11–16). A possible link between lipids and AMD has been suggested, and, recently, high-dose statins were reported to lead to resolution of signs of AMD and vision improvement (17, 18). However, the relationship between lipids and AMD has been inconsistently documented, with most studies only examining conventional measures of plasma lipids [i.e., total cholesterol, triglycerides (TGs), HDL-C and LDL-C] and AMD. Plasma lipids are carried on a heterogenous group of lipoproteins, variations in the size and density of which can alter lipid function. Importantly, within the retina, there is evidence that a sophisticated system of cholesterol uptake, intracellular trafficking, storage, and elimination utilizing lipoproteins as intermediates plays an important role in retinal physiology (19–21). Lipoproteins derived from plasma have also been implicated as the major upstream source of fatty acids within Bruchs’ membrane and provide an energy source to the retina (19, 22, 23), in addition to performing important functional roles in the transport of C, vitamin E, lutein, and zeaxanthin for use by photoreceptors (24, 25). Detailed analyses of lipoprotein profiles have provided important insights into the pathogenesis of other chronic diseases, including cardiovascular disease, insulin resistance, and diabetic retinopathy, in which endothelial dysfunction and atherosclerosis have been implicated (26–28). Thus, dysregulation in lipid metabolism, possibly affecting lipoproteins, but not necessarily captured by conventional plasma lipid measures, may also play a significant role in the pathogenesis of AMD.

In further support of this hypothesis are recent studies that show variants in genes involved in lipid metabolism, including hepatic lipase (*LIPC*), cholesterylester transfer protein (*CETP*), and ATP-binding cassette transporter A1 (*ABCA1*), confer increased risk of AMD (29–32). Animal studies have confirmed that the reverse cholesterol pathway regulated by ABC transporters may be critical in the development of a choroidal neovascularization (15). The proteins encoded by most of these cholesterol-related genes have also been immunolocalized to the retina (21). We recently completed a genome-wide association study in East Asian AMD and have identified a missense mutation (D442G) in the *CETP* gene that is associated with elevated HDL-C and an increased risk of nAMD. *CETP* mediates the transfer of cholesteryl ester from HDL to LDL (33). CETP deficiency generates enlarged HDL particles containing an excess of cholesteryl esters, which are thought to be dysfunctional and incapable of reverse cholesterol transport (34, 35). How genetic variants at the *CETP* locus influence or modulate the relationship between plasma lipids, lipoproteins, and AMD is unknown.

To address these major gaps, we examined the association between lipoprotein phenotype (size and distribution) and AMD. We also explored the possibility that the *CETP* D442G mutation may mediate or modulate these associations. Our hypothesis is that patients with AMD have abnormal lipid metabolism, which may give rise to abnormal lipoprotein profile characterized by an increase in large HDL particles, which are inefficient carriers of cholesterol.

## METHODS

### Study design and participants

We performed a case-control study utilizing serum from 193 participants of Chinese ethnicity with nAMD enrolled in a prospective clinical cohort study, the Asian AMD Phenotyping Study; and 184 subjects with early AMD and 289 age and gender-matched controls free of AMD enrolled in the Singapore Chinese Eye Study. Both studies were approved by the Singhealth Institutional Review Board and were conducted in accordance with the Declaration of Helsinki (protocol nos. R697/47/2009 and R498/47/2006). Detailed methodology of both studies has been published previously (36–42), and all participants provided written informed consent (additional information is available in supplemental data).

The Asian AMD Phenotyping Study prospectively recruited a consecutive series of treatment-naıve Asian patients with exudative maculopathy secondary to nAMD from the retinal clinic of the Singapore National Eye Centre since March 1, 2010, and is still ongoing (32–37). To facilitate comparison of potential risk factors, the Asian AMD Phenotyping study adopted methods modeled after the Singapore Epidemiology of Eye Disease program, which enrolled more than 10,000 participants by using standardized methodology and photographic grading of AMD and risk factor assessment. The Singapore Chinese Eye Study is part of the Singapore Epidemiology of Eye Disease program and is a population-based cohort study of major eye diseases in urban Chinese adults ranging from 40 to 80 years of age residing in Singapore (42). Subjects were selected from a computer-generated list provided by the Singapore Ministry of Home Affairs, using an age-stratified (by 10 year age groups) random sampling method. The study took place between 2009 and 2011. A total of 3,353 Chinese persons were eligible, and 72.8% participated in the study. Subjects with early AMD and age- and gender-matched controls without AMD were selected from this cohort.

### Clinical evaluation

#### nAMD cases.

Each patient was examined at baseline according to a standardized protocol derived in part from the Singapore Chinese Eye Study (42, 43). The examination procedures included measurements of height, weight, blood pressure, and pulse rate, followed by a comprehensive ocular examination (visual acuity, dilated fundus examination, color fundus photography, fluorescein angiography, indocyanine green angiography, and spectral domain optical coherence tomography). nAMD was diagnosed clinically and confirmed by ocular imaging results, which were graded by retinal specialists.

#### Early AMD cases and non-AMD controls.

**Fig. 1.**
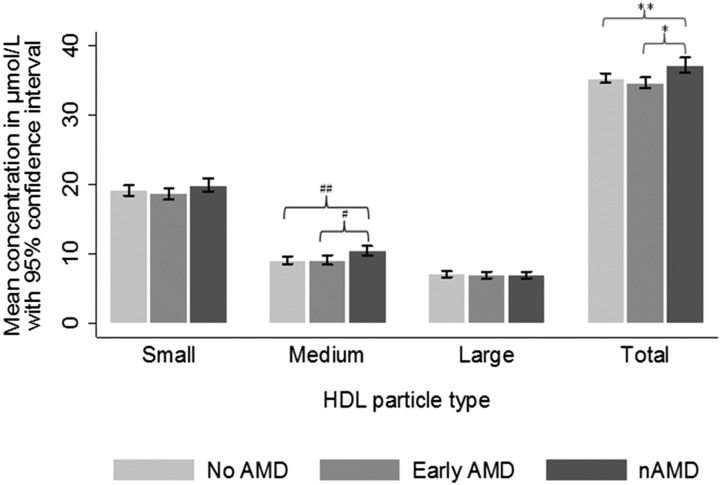
Mean serum concentration of HDL particles in participants with no AMD, early AMD, and nAMD. Participants with nAMD had significantly higher concentration of HDL compared with participants with early AMD (37.2 vs. 34.7 µmol/l), and compared with controls (37.2 vs. 35.3 µmol/l). The increase in HDL particles was mainly driven by an excess of medium-sized particles in participants with nAMD, which was significantly higher compared with participants with early AMD (10.4 vs. 9.1 µmol/l) and compared with controls (10.4 vs. 9.1 µmol/l). * *P* < 0.001; ** *P* = 0.003; ^#^*P* = 0.026; ^##^*P* = 0.009.

All participants had an interview, systemic examination and laboratory investigations to determine socioeconomic, ocular, and systemic risk factors. We used a digital fundus camera to capture color photographs of each eye after pupil dilation. Photograph of at least one eye of sufficient quality for assessment of AMD status was available in 3,312 participants (98.8%). The Centre for Vision Research, University of Sydney, performed AMD grading using a modification of the Wisconsin Age-Related Maculopathy Grading System (43), which defines early AMD as either soft indistinct or reticular drusen or both soft, distinct drusen plus retinal pigment epithelium (RPE) abnormalities. Early AMD cases and age- and gender-matched subjects free of any stage of AMD were selected as controls for this study (**Fig. 1**).

#### Medical and drug history.

A detailed interviewer-administered questionnaire was used to collect information about medical history (including hypertension, diabetes, angina, myocardial infarction, and stroke), medication including lipid-altering drugs, and cigarette smoking (defined as current, past, and never) in participants of both studies. The questionnaire was administered in English or translated into Chinese (Mandarin) and back-translated into English.

### Lipids and lipoproteins

In both cohorts, a nonfasting venous blood sample was collected at baseline. Lipid biochemistry was performed by standard automated methods at the Biochemistry Department at the Singapore General Hospital, which utilized a Beckman Coulter ExC800 automated chemistry analyzer. Total cholesterol, HDL-cholesterol, TGs, and LDL-cholesterol were analyzed based on homogenous enzymatic colorimetry assay using 500 μl of serum. Serum and plasma were extracted and stored at −80°C prior to DNA extraction.

Lipoprotein subclass levels and mean particle sizes were determined for all participants by NMR spectroscopy at LipoScience Inc. (Raleigh, NC) as previously described (26, 28), by using stored serum. Each NMR measurement produces the concentrations of three subclasses of VLDL, LDL, and HDL particles each. From the subclass levels, the weighted-mean VLDL, LDL, and HDL particle sizes (nanometers in diameter) and particle concentrations were calculated. Lipoprotein subclasses were grouped as large LDL (21.3–23.0 nm), intermediate LDL (19.8–21.2 nm), small LDL (18.3–19.7 nm), large HDL (8.8–13.0 nm), medium HDL (8.2–8.8 nm), and small HDL (7.3–8.2 nm).

### *CETP* genotyping and measurement

*CETP* D442G (rs2303790) genotype was determined from the existing Illumina Human OmniExpress genotyping or by using Taqman allelic discrimination probes (Applied Biosystems). The genotyped SNP rs2303790 was within Hardy-Weinberg Equilibrium in health controls (*P* = 0.190).

CETP concentration was determined by using a commercially available human cholesteryl ester transfer protein ELISA Kit (CSB-E08567h, Cusabio), which offers a detection range of 0.195–50 ng/ml. Serum samples (100 µl) were used for the assay following dilution and preparation instructions supplied by the vendor.

### Statistical analyses

All baseline and lipoprotein profile characteristics (LPCs) of study participants were summarized and compared between the three groups of study participants, namely, nAMD cases, early AMD cases, and age- and gender-matched controls. ANOVA using an F-test was conducted to compare the means of continuous variables, whereas a chi-squared or Fisher’s exact test was used to compare the proportional distribution of categorical variables between the three groups. Pairwise comparisons of the mean of each LPC were also performed by using Tukey’s honest significant difference test for multiple comparisons. For assessing crudely the presence of a linear trend relating the mean of each LPC with increasing AMD severity (from no AMD to early AMD to nAMD), an F-test of the linear orthogonal polynomial contrast was conducted, treating AMD severity as an equally spaced ordinal variable in a regression model.

D442G mutation carrier status was dichotomized, with individuals carrying at least one mutant allele classified as mutation carriers. The mean of each LPC between D442G mutation carriers and noncarriers was compared in nAMD cases and controls separately with a linear regression of the LPC against mutation status, adjusting for potential confounders of the relationship—age, gender, BMI, current smoking status, lipid-lowering medication, hypertension, diabetes, myocardial infarction, and stroke history. Because the mutation was very rare in the general population (∼3%), the original sample of age- and gender-matched controls had to be enriched with additional carriers of the mutation, with the augmented sample used only in this part of the analysis.

Each LPC was hereafter standardized to its distribution within age- and gender-matched controls for ease of comparing their effect sizes. To examine whether there was an independent association between each standardized LPC and the risk of nAMD or early AMD, as compared against the comparably aged normal cohort, a logistic regression model that adjusted for the abovementioned potential confounders was used for each outcome. A cumulative proportional odds model was also fitted to assess whether change in each LPC was more generally associated with the risk of presenting with a more severe stage of AMD, here regarding AMD status as a three-level ordinal outcome—the results were by and large consistent with the effect estimates obtained from the separate logistic regression models and not presented for brevity. Whether the effect of each LPC on nAMD risk was influenced by the D442G mutation was lastly investigated by adding D442G mutation status as a covariate to the logistic regression models. Expanded models allowing for interaction between each LPC and D442G mutation status were also studied to assess whether the said effects differed between mutation carriers and noncarriers. Where evidence of interaction was found, estimates of the effect of the LPC on nAMD risk were presented separately in carriers and noncarriers of the mutation.

All estimated effects derived from logistic regression models were presented as odds ratios (ORs) per SD increase in each LPC. Two-sided Wald tests and *t*-tests were used to test the coefficients of interest in logistic and linear regression models, respectively, with statistical significance concluded at *P* < 0.05. Ninety-five percentage confidence intervals were presented for all reported effect estimates. The statistical packages Stata 12 and R (version 3.2.1) were used for the analyses.

### Patient involvement

No patients were involved in setting the research question or the outcome measures. Patients were not involved in developing plans for recruitment, design, or implementation of the study or the interpretation and presentation of results. The results of the research will not be disseminated to the individual patient.

## RESULTS

### Lipoprotein phenotypes in nAMD cases, early AMD cases, and controls

The baseline characteristics of participants are summarized in **Table 1**. Apart from slightly younger age in the early AMD group, there were no statistically significant differences in gender, BMI, smoking status, or history of hypertension, diabetes, myocardial infarction, or stroke between the three groups of patients. The proportion of subjects on lipid medication was similar in all three groups. The D442G minor frequency allele was present in 15.0% of the late AMD group, 2.2% of the early AMD group, and 2.8% of controls. As expected, the presence of the D442G polymorphism was associated with increased risk of late AMD [OR = 6.1 (95% CI 2.7–13.7), *P* < 0.001]. Conventional lipid biochemistry showed similar levels of HDL-C, LDL-C, and TGs between the three groups.

**TABLE 1.** Baseline characteristics of controls, early AMD cases, and late AMD cases

Baseline variable	No AMD (n = 289)	Early AMD (n = 178)	nAMD (n = 193)	*P**^a^*
Age (years), mean (SD)	67.4 (9.3)	65.2 (9.7)	67.5 (10.0)	0.036
Male, %	59.9	53.9	61.1	0.316
BMI (kg/m^2^), mean (SD)	23.7 (3.8)	23.4 (3.7)	24.0 (4.0)	0.344
Current smoker, %	13.1	10.1	18.3	0.091
Self-reported hypertension, %	43.9	49.4	53.3	0.153
Self-reported diabetes, %	14.9	13.5	12.5	0.776
Self-reported myocardial infarction, %	6.2	6.2	5.3	0.919
Self-reported stroke, %	3.1	1.7	2.0	0.670
On lipid medication, %	35.9	33.3	35.8	0.838
Carrier of *CETP* D442G variant, %	2.8	2.2	15.0	<0.001
Conventional lipid biochemistry				
HDL-C (mmol/l), mean (SD)	1.3 (0.4)	1.3 (0.4)	1.4 (0.3)	0.214
LDL-C (mmol/l), mean (SD)	3.1 (0.9)	3.2 (0.8)	3.0 (1.0)	0.136
TG (mmol/l), mean (SD)	1.8 (1.1)	1.8 (1.2)	1.8 (0.9)	0.819

a ANOVA using an F-test was performed to compare the mean of continuous variables, whereas a chi-squared or Fisher’s exact test was used to compare proportions for categorical variables. Fisher’s exact test was used when one or more cells had a sample size ≤5.

Results of detailed lipoprotein profile are summarized in **Tables 2** and **3**. Although HDL-C, LDL-C, and TGs were similar between nAMD cases and controls, more detailed examination of the lipoprotein particle revealed significant differences between controls and patients with early or late AMD. First, nAMD cases had lower plasma levels of apo A-I (Apo-A1). Among the TG-rich lipoproteins, nAMD cases had lower concentrations of VLDL and chylomicron particles. These differences were attributable to lower concentrations of medium VLDL and chylomicron particles. Small VLDL particle concentration did not differ between groups. These lower levels of VLDL and chylomicron levels were associated with higher levels of IDL, but lower levels of large LDL particles. In relation to HDL particles, nAMD cases had a higher HDL particle concentration, which was largely due to an excess of medium HDL particles. Many of these associations showed a dose-dependent association with early AMD cases and nAMD cases (**Tables 4** and **5** and Fig. 1). In a supplementary analysis that included only subjects who were not taking any lipid-lowering medications, the associations related to ApoA1, LDL, VLDL, and chylomicrons and IDL remained statistically significant, whereas the associations related to HDL remained in the same direction, although not reaching statistical significance.

**TABLE 2.** Comparison of lipoprotein profiles in controls, early AMD cases and nAMD cases

	Mean (SD)			*P**^a^*	Pairwise comparison of means*^a^*			*P* for linear trend*^b^*
	No AMD (n = 289)	Early AMD (n = 178)	nAMD (n = 193)		Early AMD versus no AMD	nAMD versus no AMD	nAMD versus early AMD	
Total particle concentration by subclass								
ApoA1, mg/ld.	155.8 (26.7)	154.6 (23.6)	145.3 (45.5)	0.002	0.928	0.002	0.017	<0.001
HDL, µmoll/l	35.3 (6.0)	34.8 (5.2)	37.2 (7.4)	<0.001	0.618	0.003	<0.001	0.001
LDL, nmol/l	1,320.8 (439.6)	1,222.4 (360.8)	1,236.5 (416.9)	0.019	0.034	0.070	0.942	0.029
VLDL and chylomicron lipoprotein, nmol/l	73.2 (30.5)	67.4 (28.4)	60.6 (23.2)	<0.001	0.083	<0.001	0.049	<0.001
Lipoprotein size distribution by subclass								
HDL, µmol/l								
Large	7.1 (3.8)	6.9 (3.6)	6.9 (3.5)	0.838	0.878	0.868	1.000	0.614
Medium	9.1 (5.0)	9.2 (4.5)	10.4 (5.0)	0.008	0.951	0.008	0.046	0.003
Small	19.1 (6.4)	18.6 (5.7)	19.9 (6.4)	0.131	0.648	0.383	0.114	0.187
IDL, nmol/l								
IDL	110.7 (79.6)	114.7 (82.6)	155.6 (110.6)	<0.001	0.891	<0.001	<0.001	<0.001
LDL, nmol/l								
Large	581.2 (264.5)	554.0 (240.4)	448.3 (269.6)	<0.001	0.515	<0.001	<0.001	<0.001
Small	628.8 (427.7)	553.7 (343.1)	632.6 (375.5)	0.084	0.111	0.994	0.129	0.917
VLDL and chylomicron, nmol/l								
Large	6.6 (6.3)	6.1 (5.9)	5.2 (3.8)	0.030	0.639	0.022	0.270	0.008
Medium	30.8 (19.4)	25.4 (16.2)	17.5 (12.1)	<0.001	0.002	<0.001	<0.001	<0.001
Small	35.8 (17.9)	36.0 (17.3)	37.9 (16.5)	0.394	0.989	0.392	0.555	0.192
Mean particle sizes								
HDL particle size, nm	9.2 (0.5)	9.3 (0.5)	9.3 (0.5)	0.064	0.371	0.057	0.691	0.022
LDL particle size, nm	20.9 (0.6)	21.0 (0.6)	20.8 (0.6)	0.056	0.292	0.480	0.044	0.248
VLDL particle size, nm	49.8 (7.5)	49.8 (8.2)	50.5 (7.6)	0.529	0.999	0.542	0.640	0.292

a ANOVA using an F-test was performed to compare mean lipoprotein levels between the three stages of AMD, and Tukey’s honest significant difference test was used for multiple pairwise comparisons.

b Evidence of trend was assessed by conducting an F-test of the linear orthogonal polynomial contrast in a regression model of lipoprotein level against AMD stage as a three-level ordinal variable.

**TABLE 3.** Comparison of lipoprotein profiles in controls, early AMD cases and nAMD cases after excluding subjects taking lipid-lowering medications

	Mean (SD)			*P*-value*^a^*	Pairwise comparison of means*^a^*			P-value for linear trend*^b^*
	No AMD (n = 184)	Early AMD (n = 121)	nAMD (n = 95)		Early AMD versus no AMD	nAMD versus no AMD	nAMD versus early AMD	
Total particle concentration by subclass								
ApoA1, mg/dl	154.4 (27.0)	153.3 (20.0)	137.1 (45.8)	<0.001	0.953	<0.001	<0.001	<0.001
HDL, µmol/l	34.8 (6.0)	33.9 (4.7)	35.6 (7.0)	0.098	0.422	0.468	0.081	0.240
LDL, nmol/l	1,426.5 (457.7)	1,239.5 (377.5)	1,316.9 (449.9)	<0.001	<0.001	0.113	0.387	0.046
VLDL and chylomicron lipoprotein, nmol/l	74.2 (31.0)	66.2 (29.4)	60.2 (23.9)	<0.001	0.049	<0.001	0.284	<0.001
Lipoprotein size distribution by subclass								
HDL, µmol/l								
Large	7.0 (3.9)	7.0 (3.3)	6.9 (3.5)	0.971	0.999	0.969	0.981	0.812
Medium	9.0 (5.2)	9.2 (4.6)	10.1 (5.7)	0.210	0.956	0.195	0.370	0.084
Small	18.8 (6.6)	17.7 (5.4)	18.6 (5.7)	0.333	0.324	0.984	0.634	0.865
IDL, nmol/l								
IDL	120.6 (81.2)	120.7 (89.2)	165.3 (111.0)	<0.001	1.000	<0.001	0.001	<0.001
LDL, nmol/l								
Large	637.7 (286.7)	574.0 (252.4)	495.2 (281.9)	<0.001	0.121	<0.001	0.095	<0.001
Small	668.2 (473.4)	543.8 (347.3)	656.4 (406.1)	0.034	0.034	0.973	0.129	0.824
VLDL and chylomicron, nmol/l								
Large	6.8 (6.7)	6.1 (6.0)	5.2 (3.8)	0.078	0.514	0.065	0.499	0.025
Medium	31.0 (20.0)	24.9 (16.4)	17.3 (11.4)	<0.001	0.008	<0.001	0.004	<0.001
Small	36.4 (18.3)	35.2 (18.7)	37.7 (17.8)	0.610	0.846	0.836	0.581	0.569
Mean particle sizes								
HDL particle size, nm	9.2 (0.5)	9.3 (0.5)	9.4 (0.5)	0.031	0.134	0.045	0.840	0.017
LDL particle size, nm	20.9 (0.6)	21.0 (0.6)	20.9 (0.6)	0.169	0.216	0.977	0.241	0.837
VLDL particle size, nm	49.4 (7.8)	49.8 (8.3)	50.3 (7.8)	0.647	0.907	0.622	0.877	0.354

a ANOVA using an F-test was performed to compare mean lipoprotein levels between the three stages of AMD, and Tukey’s honest significant difference test was used for multiple pairwise comparisons.

b Evidence of trend was assessed by conducting an F-test of the linear orthogonal polynomial contrast in a regression model of lipoprotein level against AMD stage as a three-level ordinal variable.

**TABLE 4.** Relationship between the nAMD and early AMD with lipoprotein profiles

	Early AMD versus controls		nAMD versus controls	
	OR*^a^* (95% CI)	*P**^b^*	OR*^a^* (95% CI)	*P**^b^*
Conventional lipid biochemistry				
HDL-C, mmol/l	1.05 (0.84–1.31)	0.664	1.33 (1.03–1.71)	0.027
LDL-C, mmol/l	1.07 (0.86–1.35)	0.536	0.87 (0.69–1.11)	0.265
TG, mmol/l	0.95 (0.77–1.18)	0.639	1.03 (0.78–1.35)	0.849
Total particle concentration by subclass				
ApoA1, mg/dl	0.86 (0.68–1.04)	0.105	0.68 (0.57–0.82)	<0.001
HDL particles (total,) µmol/l	0.79 (0.63–1.00)	0.053	1.26 (1.01–1.57)	0.039
LDL particles (total), nmol/l	0.71 (0.55–0.90)	0.005	0.80 (0.63–1.02)	0.076
VLDL and chylomicron particles (total), nmol/l	0.84 (0.68–1.04)	0.105	0.54 (0.41–0.71)	<0.001
Lipoprotein size distribution by subclass				
HDL				
Large	0.88 (0.70–1.11)	0.287	0.92 (0.71–1.18)	0.495
Medium	0.98 (0.80–1.21)	0.877	1.24 (1.01–1.52)	0.039
Small	0.90 (0.72–1.11)	0.321	1.07 (0.86–1.34)	0.523
IDL				
IDL particles	1.06 (0.86–1.29)	0.595	1.68 (1.38–2.04)	<0.001
LDL				
Large	0.84 (0.68–1.04)	0.116	0.57 (0.44–0.72)	<0.001
Small	0.78 (0.62–0.99)	0.041	0.98 (0.78–1.23)	0.865
VLDL and chylomicron				
Large	0.90 (0.72–1.12)	0.351	0.63 (0.47–0.86)	0.003
Medium	0.74 (0.59–0.93)	0.009	0.30 (0.21–0.43)	<0.001
Small	1.03 (0.84–1.27)	0.754	1.10 (0.89–1.37)	0.356
Mean particle sizes				
HDL particle size	1.11 (0.89–1.38)	0.355	1.36 (1.08–1.71)	0.010
LDL particle size	1.11 (0.90–1.37)	0.341	0.93 (0.74–1.17)	0.559
VLDL particle size	0.99 (0.81–1.21)	0.904	1.04 (0.84–1.30)	0.713

a The OR relating a one SD increase in lipoprotein to early/late stage AMD was estimated from separate multiple logistic regression models adjusted for age, gender, BMI, smoking status, average axial length, lipid-lowering medication, self-reported hypertension, diabetes, myocardial infarction, and stroke.

b *P*-values were derived from Wald tests of the corresponding logistic regression coefficient relating each lipoprotein to early/late-stage AMD.

**TABLE 5.** Relationship between the nAMD and early AMD with lipoprotein profiles after excluding subjects taking lipid-lowering medications

	Early AMD versus controls		nAMD versus controls	
	OR*^a^* (95% CI)	*P^b^*	OR*^a^* (95% CI)	*P**^b^*
Conventional lipid biochemistry				
HDL-C, mmol/l	1.12 (0.84–1.50)	0.431	1.54 (1.11–2.14)	0.010
LDL-C, mmol/l	0.89 (0.67–1.16)	0.395	0.85 (0.65–1.12)	0.252
TG, mmol/l	0.96 (0.75–1.24)	0.768	0.97 (0.68–1.38)	0.847
Total particle concentration by subclass				
ApoA1, mg/dl	0.918 (0.67–1.23)	0.536	0.64 (0.51–0.81)	<0.001
HDL particles (total,) µmol/l	0.81 (0.61–1.09)	0.166	1.19 (0.90–1.59)	0.227
LDL particles (total), nmol/l	0.67 (0.42–0.78)	<0.001	0.73 (0.55–0.99)	0.040
VLDL and chylomicron particles (total), nmol/l	0.73 (0.56–0.96)	0.027	0.51 (0.36–0.72)	<0.001
Lipoprotein size distribution by subclass				
HDL				
Large	0.97 (0.72–1.31)	0.848	1.10 (0.80–1.52)	0.554
Medium	1.03 (0.80–1.32)	0.845	1.25 (0.98–1.60)	0.073
Small	0.83 (0.63–1.10)	0.193	0.89 (0.66–1.194)	0.435
IDL				
IDL particles	1.03 (0.81–1.31)	0.807	1.64 (1.28–2.10)	<0.001
LDL				
Large	0.77 (0.60–0.98)	0.036	0.61 (0.46–0.81)	<0.001
Small	0.69 (0.52–0.93)	0.013	0.89 (0.67–1.18)	0.422
VLDL and chylomicron				
Large	0.89 (0.68–1.16)	0.378	0.64 (0.44–0.93)	0.020
Medium	0.71 (0.54–0.95)	0.020	0.30 (0.19–0.47)	<0.001
Small	0.88 (0.68–1.14)	0.335	1.01 (0.78–1.32)	0.913
Mean particle sizes				
HDL particle size	1.29 (0.98–1.70)	0.070	1.75 (1.28–2.40)	<0.001
LDL particle size	1.22 (0.94–1.60)	0.140	1.10 (0.82–1.49)	0.520
VLDL particle size	1.06 (0.83–1.35)	0.652	1.10 (0.84–1.45)	0.477

a The OR relating a one SD increase in lipoprotein to early/late stage AMD was estimated from separate multiple logistic regression models adjusted for age, gender, BMI, smoking status, average axial length, lipid-lowering medication, self-reported hypertension, diabetes, myocardial infarction, and stroke.

b *P*-values were derived from Wald tests of the corresponding logistic regression coefficient relating each lipoprotein to early/late-stage AMD.

### Influence of CETP D442G polymorphism on lipoprotein phenotype

After multivariable adjustment, we found that for each SD increase in HDL particles, a 26% increase in nAMD risk was observed (*P* = 0.039). The associations between HDL and increased risk of nAMD observed remained significant after adjustment for the presence of the D442G polymorphism at the *CETP* locus (analysis performed but not shown). To further evaluate the effect of *CETP* D442G polymorphism on lipoprotein phenotypes independent of AMD, we measured the lipoprotein profile in a further 113 non-AMD subjects who were carriers of the D442G mutation (total of 121 non-AMD subjects carrying the D442G mutation) and compared them with 276 non-AMD controls without the mutation (**Table 6**). Among these non-AMD controls, presence of the *CETP* D442G mutation was associated with significantly reduced level of CETP protein (432.9 vs. 569.2 ng/ml, *P* < 0.001) and elevated HDL-C levels (1.5 vs. 1.3 mmol/l, *P* < 0.001), as expected. Comparison of lipoprotein particle concentrations showed that D442G mutation was associated with lower medium VLDL, higher small VLDL, and larger LDL particle size. There was no significant difference in any of the lipoprotein particle concentration in AMD cases according to D442G mutation status.

**TABLE 6.** Lipoprotein profiles in controls and late AMD cases according to CETP D442G status

	Mean (SD)				*P**^a^*	*P**^a^*
	Controls		Late AMD		Controls	Late AMD
	Without D442G (n = 276)	With D442G (n = 121)	Without D442G (n = 164)	With D442G (n = 29)	Without versus with D442G	Without versus with D442G
CETP concentration, ng/ml						
	569.2 (232.2)	432.9 (189.5)	538.1 (302.7)	528.9 (342.7)	<0.001	0.773
Conventional lipid biochemistry						
HDL-C, mmol/l	1.3 (0.4)	1.5 (0.5)	1.4 (0.3)	1.5 (0.4)	<0.001	0.497
LDL-C, mmol/l	3.1 (0.9)	3.4 (0.9)	3.0 (1.0)	2.9 (1.0)	0.247	0.892
TG, mmol/l	1.8 (1.1)	1.8 (1.2)	1.9 (1.0)	1.6 (0.7)	0.515	0.509
Total particle concentration by subclass						
ApoA1, mg/dl	155.1 (26.4)	160.3 (25.0)	144.2 (47.0)	151.4 (35.8)	0.529	0.968
HDL, µmol/l	35.1 (6.0)	36.9 (6.2)	37.1 (7.4)	37.9 (7.2)	0.379	0.272
LDL, nmol/l	1,326.5 (446.0)	1,316.4 (428.2)	1,264.9 (425.3)	1,076.4 (328.6)	0.387	0.487
VLDL and chylomicron lipoprotein, nmol/l	73.3 (30.4)	71.6 (26.8)	61.3 (24.0)	56.4 (17.2)	0.674	0.303
Lipoprotein size distribution by subclass						
HDL						
Large	7.0 (3.7)	7.9 (3.9)	6.7 (3.2)	8.2 (4.8)	0.092	0.308
Medium	9.0 (5.0)	9.3 (4.7)	10.4 (5.1)	10.7 (4.4)	0.728	0.255
Small	19.2 (6.3)	19.8 (5.7)	20.1 (6.6)	19.0 (4.8)	0.710	0.512
IDL						
	110.9 (80.2)	124.4 (92.2)	157.5 (112.0)	145.2 (103.5)	0.111	0.726
LDL						
Large	578.2 (268.4)	633.2 (269.7)	443.1 (260.5)	477.6 (320.0)	0.294	0.319
Small	637.5 (430.5)	558.8 (431.3)	664.2 (386.2)	453.6 (243.8)	0.069	0.122
VLDL and chylomicron						
Large	6.7 (6.4)	6.7 (5.5)	5.2 (3.9)	5.1 (3.3)	0.973	0.292
Medium	31.0 (19.5)	23.5 (16.0)	18.1 (12.6)	14.4 (7.7)	0.003	0.191
Small	35.6 (17.9)	41.4 (18.7)	38.0 (16.6)	36.9 (15.9)	<0.001	0.773
Mean particle size						
HDL particles, nm	9.2 (0.5)	9.3 (0.5)	9.3 (0.5)	9.4 (0.5)	0.088	0.115
LDL particles, nm	20.9 (0.6)	21.1 (0.7)	20.8 (0.6)	21.0 (0.6)	0.003	0.346
VLDL and chylomicron, nm	49.8 (7.6)	50.9 (7.8)	50.4 (7.5)	51.6 (8.1)	0.192	0.450

a *P*-values were derived from *t*-tests of the adjusted difference in mean lipoprotein level between mutation carriers and noncarriers (for cases and controls separately) in a multiple linear regression model adjusted for age, gender, BMI, smoking status, average axial length, lipid-lowering medication, self-reported hypertension, diabetes, myocardial infarction, and stroke.

We found a significant interaction between D442G mutation and HDL-particle concentration on the risk of AMD (**Table 7**). In noncarriers of the D442G allele, higher levels of both total HDL and medium HDL particle numbers were associated with increased risk of nAMD. In carriers of the D442G allele, although there was a trend toward higher concentration of these particles with lower nAMD risks, the results were not statistically significant.

**TABLE 7.** Interaction between HDL particle concentration and CETP D442G mutation on the risk of nAMD

	Group without D442G mutation (n = 440)	Group with D442G mutation (n = 37)	Interaction coefficient*^b^*
Number with AMD	164	29	—
Percentage with AMD (%)	37.3	78.4	—
Effect of total HDL particle concentration on AMD			
OR*^a^* per SD increase in total HDL particle concentration	1.32 (1.06–1.65) *P* = 0.014	0.44 (0.16–1.20) *P* = 0.107	0.33 (0.12–0.93) *P* = 0.036
Effect of medium-sized HDL particle concentration on AMD:			
OR*^a^* per SD increase in medium-sized HDL particle concentration	1.30 (1.06–1.61) 0.014	0.34 (0.10–1.18) 0.088	0.26 (0.07–0.92) 0.037

a The OR relating increase in each lipoprotein to late-stage AMD by mutation carrier status was estimated from a logistic regression model that included an interaction term between the lipoprotein and mutation status, in addition to their primary effects, age, gender, BMI, smoking status, average axial length, lipid-lowering medication, self-reported hypertension, diabetes, myocardial infarction, and stroke.

b The interaction coefficient estimates the ratio of the OR in mutation carriers to that in noncarriers in the abovementioned model, with the *P*-value derived from a Wald test of the said coefficient.

## DISCUSSION

There is a strong biological rationale and underlying hypothesis that lipid dysregulation may be involved in the pathogenesis of AMD. First, genetic studies have reported variants in several C-related genes to confer increased risk of AMD (29–32). Second, there is biochemical evidence to suggest that intraretinal lipid transport is facilitated by proteins similar to those in systemic lipid metabolism (11–13). Third, patients with AMD have an increased risk of atherosclerosis and coronary artery disease (CAD), and, likewise, atherosclerosis and cardiovascular disease are major risk factors for development of AMD (44–48). Fourth, studies in primates (49, 50) and rodents (15, 16) have demonstrated that oxidized lipids may accumulate in the retina and promote angiogenesis directly or via impaired macrophage cholesterol efflux, although we acknowledge that the relevance of these animal models to human AMD is uncertain.

Despite the strong biological rationale, the relationship between lipid metabolism and AMD has been inconsistently documented in clinical studies, with most studies examining only the conventional plasma lipid measures of TC, TG, HDL-C, and LDL-C levels. In the current study, we hypothesized that patients with AMD may have abnormal lipid metabolism that may be reflected as changes in the subtype of lipoprotein particles in the blood. We demonstrated that patients with nAMD and early AMD exhibit marked differences in many lipoprotein subclasses tested compared with controls, characterized by higher concentrations of HDL particles, particularly medium-sized particles, and IDL particles, and lower concentrations of Apo A-1, VLDL, and chylomicron particles (Tables 2–5). These findings are in line with the hypothesis that tissue lipids may represent therapeutic targets for the treatment and prevention of AMD.

Comparing and contrasting our observations in AMD with the relationship between lipoprotein profile and CAD provides insights into mechanisms. Although AMD has previously been associated with CAD, the pattern of associations of lipoprotein with AMD demonstrated in this study is not similar to that for CAD. Specifically, high VLDL and LDL particle concentrations are usually associated with increased risk of CAD, whereas we found that nAMD was associated with lower VLDL particle concentration. Conversely, total and large HDL particle concentrations are consistently inversely associated with CAD risk (51), whereas in our AMD cases, the association was positive. On the other hand, raised IDL observed in nAMD patients may be a shared risk factor with CAD, because this is known to be atherogenic.

We recently reported on a genome-wide association study in East Asians and identified a strong association between the *CETP* D442G mutation with nAMD (per-allele OR = 1.70) (33). *CETP* mediates the transfer of cholesteryl ester from HDL to LDL. Together with hepatic lipase LIPC, these two genes are the key genetic determinants of lipoprotein sizes (52, 53). The D442G mutation is specific to Asians, with minor allele frequency of 0.03 in East Asians and <0.01 in Europeans. The mutant 442G allele is known to impair CETP function, resulting in reduced CETP mass and activity (34, 35, 54). Each copy of the dysfunctional 442G allele conferred, on average, a rise in HDL-C levels of 0.174 mmol/l. We were therefore particularly interested to investigate the potential influence of HDL particles on AMD risk. Our results demonstrated that increase in HDL particle concentration, particularly medium-sized particles, was significantly associated with nAMD.

To understand this association, we need to understand the function of HDL described in previous studies. HDL play a unique role in supporting reverse C transport, which represents a major pathway through which excess C in peripheral tissues, such as endothelium, can be removed. These particles therefore protect against effects from excess C in the peripheral tissues (55–57). Accumulation of intracellular C may also result from aging of macrophages, with features including abnormal polarization, downregulation of ABCA1, and impaired C efflux, which, in turn, can lead to increased inflammation and a proangiogenic state (15). HDL-C may also have additional antiinflammatory, antioxidant, antiaggregatory, anticoagulant, and profibrinolytic activities. However, these protective properties of HDL may vary according to the structure and function of the lipoprotein particles on which they are carried. Thus, our finding that risk of AMD was not associated with HDL-C, but was associated with the number of particles (particularly of medium-sized HDL particles), is interesting, but not altogether surprising. In addition, our finding of reduced concentration of Apo A-1 in patients with nAMD further supports that the structure and function of HDL particles may be altered in AMD. Apo-A1 is a major apolipoprotein in HDL and one that is thought to mediate many of the protective effects of HDL. This suggests that the number of HDL particles, while increased, may be depleted of Apo-A1, adversely affecting their ability to facilitate cholesterol efflux. In support of this hypothesis, within the retina, Apo-A1 has been observed in the apical region of the RPE, in the interphotoreceptor matrix of rod photoreceptors, choroid, and neuroretina in monkey retina. The apical location within the RPE suggests that Apo-A1 may be secreted by RPE into the interphotoreceptor matrix (21). One proposed mechanism in which RPE can remove oxidized lipids arising in the photoreceptor outer segments may be through transfer of the lipids into their endogenous Apo-A1- and ApoE-containing HDL-like particles, which are, in turn, transported by ABCA1 out of the RPE. However, these endogenous HDL-like particles remain to be identified.

Although we confirmed our a priori hypothesis that perturbation in HDL metabolism would be the main changes seen in AMD, we were somewhat surprised to find that VLDL and chylomicron lipoprotein concentration was markedly reduced in subjects with AMD. In the retina, studies have demonstrated that LDL is the major carrier of cholesterol from the systemic circulation into RPE and neurosensory retina (58–60). Although the function of these lipids within the retina remain to be elucidated, recent findings using VLDL receptor (VLDLR) KO mice suggest that lipid β-oxidation is an important energy source for the retina. Fuel shortage resulting from reduced uptake of TG-derived fatty acid and glucose, in turn, leads to pathological angiogenesis in the retina through stabilization of hypoxia-induced factor 1a and secretion of vascular endothelial growth factor A by photoreceptors (58–60). The relevance of the VLDLR to the pathogenesis of AMD is further supported by genetic association studies (61). The finding of markedly reduced VLDL particle concentration from the current study further supports the hypothesis that eyes with AMD require fatty acids from VLDL. It is our hypothesis that the reduced availability of VLDL results in reduced fatty acid uptake by the retinal cells, which may, in turn, result in a proangiogenic state. The mechanism leading to the low VLDL in AMD patients is unclear, but may include reduced production from the liver, increased catabolism by LPL, and increased uptake by tissues other than the eye. However, associations between variants at the *LPL* locus and increased susceptibility to AMD have so far been inconsistently described (29, 62, 63).

We explored the possibility that the presence of the D442G mutation at the *CETP* locus may explain the association between lipoprotein particle concentrations and AMD (Table 6). In controls, the presence of the 442G allele was associated with lower CETP activity and higher HDL-C, a finding that is consistent with previous studies (54, 64, 65). However, inclusion of the D442G mutation in the model did not significantly alter the association between HDL particle concentrations and increased risk of nAMD. As such, factors other than this mutation in *CETP* must play an important role in the pathogenesis of dyslipidemia associated with AMD. First, other mutations at the *CETP* locus may be important. Six polymorphisms in *CETP* have previously been described (26). Of specific relevance, the presence of the V405 allele of rs5882 has a similar, but smaller, effect to D442G mutation and is associated with higher HDL-C and reduced CETP activity. This polymorphism has been reported to be associated with polypoidal choroidal vasculopathy, a subtype of AMD common in Asians (62). Second, the effect of *CETP* mutations on AMD risk may not be mediated through HDL particles in the systemic circulation, but by a local effect in the retina. Indeed, CETP has been localized to the outer plexiform layer and the photoreceptor outer segments and interphotoreceptor matrix in monkey retina (21), suggesting that the retina has the ability to mature HDL particles locally and to transfer cholesterylester between lipoproteins. Therefore, dysfunction in CETP could lead to localized impairment of HDL maturation within the retina, resulting in impaired lipid transfer between the RPE and photoreceptors.

There are limitations in the current study. Changes demonstrated in plasma may not reflect the exact changes within the chorioretina. Future studies should aim to incorporate evaluation of cholesterol metabolism within the retina. In particular, further experiments based on human tissues will be needed to ascertain the translatability of studies in rodents and primates. Although we demonstrated marked changes in all the subclasses of lipoproteins tested, we were not able to evaluate the relative influence of different pathways. Knowledge of which pathways are the most important will be essential in guiding future therapeutic approaches. Because of the limited number of patients with nAMD in population studies, our case-control study compared subjects from a hospital-based cohort (the Asian AMD Phenotyping study) with those from a population-based study (the Singapore Chinese Eye Study). Thus, despite the carefully designed and standardized protocols between the two studies, we acknowledge that there remain inherent differences in cases and controls, and the possibility of selection bias. Approximately 35% of subjects in each AMD category was on lipid-lowering medication. However, it is unlikely that the associations reported are affected by lipid medication because we have included lipid-lowering medication as one of the variables in the multivariable models, and most of the associations remained significant, even in a subgroup analysis that included only subjects not taking lipid-lowering medications. Finally, blood samples were in nonfasting conditions. Ideally, we would have collected the samples in the fasting state. However, the controls in this study were collected as part of a much larger study to examine the prevalence and risk factors of a number of different eye diseases in the Singapore population. Multiple complex measurements related to eye disease were required, and, logistically, we were not able to study all the participants in the fasting state. For this reason, we collected the blood samples from cases in the nonfasting state as well. We believe that lipid measurements in nonfasting samples would still be relevant. Most humans spend their day in the nonfasting state, and, therefore, the nonfasting state may actually be more physiologically relevant to health and disease than the fasting state. The use of fasting lipids for predicting the risk of future disease is largely historical, and, for most purposes, measurement of lipids in the fasting state is considered preferable but not essential (66). In fact, the European Atherosclerosis Society and European Federation of Clinical Chemistry and Laboratory Medicine recently issued a statement suggesting that fasting is not essential for determination of a lipid profile in relation to predicting cardiovascular disease and making decisions on treatment. This is based on studies that have shown that nonfasting lipids are as (if not more) predictive of cardiovascular disease as fasting lipids (67, 68). This does not exclude the possibility that differences in the timing of blood sampling in relation to the last meal prior to blood sampling could bias the results. We feel that the pattern of lipoproteins associated with AMD in this instance makes it less likely that our findings merely reflect bias due to the nonfasting samples. First, TG is one of the blood lipids that is most affected by food intake and is, on average, 0.3 mmol/l higher in nonfasting samples than in fasting samples (67). We did not see any significant differences in TG levels between cases and controls. Second, postprandial changes in lipoprotein particles have been studied using NMR in more than 1,000 men and women (69). In this study, both men and women exhibited a reduction in the total LDL-particle concentration following a meal, which was associated with an increase in IDL and large LDL particles and a reduction in small LDL particles. In our study, we observed reduced total LDL particle concentration, increased IDL particle concentration, and reduced small LDL particle concentration. However, unlike the observation following a meal, when large LDL particle concentration increased, we observed a reduction in large LDL particle concentrations, which was highly statistically significant. This suggests that the differences observed between cases and controls may not relate to food intake. To further evaluate the potential effects of differences in the way samples were collected among cases and controls, we also evaluated the lipoprotein particle concentrations in 17 subjects from the Singapore Chinese Eye Study who had nAMD and had blood available that had been collected at the same time and using the same protocol as the controls utilized in this study. The results from these 17 subjects are presented alongside our findings in supplemental Table S1. The low concentrations of medium VLDL and large LDL particles, accompanied by high concentrations of IDL particles that we observed in cases compared with controls were also observed in these 17 cases derived from the Singapore Chinese Health Study (the study from which the controls were derived). In relation to HDL, there was a relatively larger proportion of large HDL particles in these cases compared with controls (as we saw with the other cases), reflected as a larger HDL particle size. However, the concentrations of the individual HDL species were dissimilar from the other cases. Chylomicron concentrations were also different in these 17 cases compared with the cases derived from the Asian AMD phenotyping study. Thus, although we are reasonably confident in our findings related to VLDL, IDL, and LDL particles, we feel that some of these studies should be repeated in a study with fasting collection of samples, which may be particularly relevant to our understanding of the role of chylomicron and HDL particles in the pathogenesis of AMD.

## CONCLUSION

To conclude, we demonstrate that altered concentration of lipoprotein particles are associated with AMD that are not captured by conventional lipid measures. We report that nAMD is associated with higher concentrations of HDL particles, particularly medium-sized HDL particles, and IDL particles, and lower concentration of VLDL and chylomicron and Apo A-1. These relationships were not mediated by the *CETP* D442G mutation. However, the associations may be modulated by the presence of this mutation. Although our study does not provide definitive proof of a causal link between dyslipidemia and AMD, it does support the relevance of the VLDLR KO mouse model of AMD to humans and raises the possibility that therapies that increase the supply of fatty acids through VLDL to the retina, or that may reduce the reliance on lipids as a fuel, could be relevant to the prevention and treatment of AMD.

## Supplementary Material

Supplemental Data
