# Supplementary material for: Plasma lipoprotein subfraction concentrations are associated with lipid metabolism and age-related macular degeneration
Source: J Lipid Res. 2017 Jul 11;58(9):1785–96. doi: 10.1194/jlr.M073684 (PMC5580892; doi:10.1194/jlr.M073684)
Supplement: Supplemental Data [file supp_58_9_1785__index.html]

Plasma lipoprotein sub-fraction concentrations are associated with Lipid Metabolism and Age-related Macular Degeneration — Plasma lipoprotein subfraction concentrations are associated with lipid metabolism and age-related macular degeneration — Supplemental Data 

# Plasma lipoprotein subfraction concentrations are associated with lipid metabolism and age-related macular degeneration

## Supplemental Data

- Supplementary Methodology and Table (.docx, 216 KB) - Supplementary Methodology and Table
