# Supplementary material for: Plasma lipoprotein subfraction concentrations are associated with lipid metabolism and age-related macular degeneration
Source: J Lipid Res. 2017 Jul 11;58(9):1785–96. doi: 10.1194/jlr.M073684 (PMC5580892; doi:10.1194/jlr.M073684)
Supplement: Supplemental Data [file 10.1194_M073684_jlr.M073684-1.docx]

**Supplementary Methodology**

**The Singapore Chinese Eye Study**

The Singapore Chinese Eye study was a population-based study conducted between 2009 and 2011. The study was conducted at the Singapore Eye Research Institute with approval from the Singhealth Institutional Review Board and in accordance with the Declaration of Helsinki. All participants provided written informed consent. Participants aged 40 to 80 years of age residing in southwest Singapore were identified from a computer-generated list provided by the Singapore Ministry of Home Affairs using an age-stratified (by 10-year age group) random sampling method. Participants’’ ethnicity was determined as indicated on the National Registration Identity card which is provided to all Singapore citizens and permanent residents. The number of eligible individuals was 4606 Chinese persons, of which 3353 (72.8%) participated. The specific aims were:

- To determine the prevalence and impact of visual impairment and of the major eye diseases threatening vision in ethnic Indian and Chinese persons living in Singapore, including early and late AMD, diabetic retinopathy, myopia, open and closed angle glaucoma, different cataract subtypes and their impact on quality of life and cognitive status, the awareness and understanding of eye diseases and utilization of eye care services.
- To compare and evaluate racial/ethnic variability in eye disease prevalence, causes, risk factors, availability of care, and impact among ethnic Indian and Chinese participants of the SICC and ethnic Malays of the SiMES participants.
- To examine the relationship of novel retinal image analysis to ocular (e.g., AMD, diabetic retinopathy, glaucoma, and myopia) and systemic disease prevalence (e.g., hypertension, diabetes, cardiovascular, and renal disease).
- To collect blood specimens and establish a DNA bio bank repository for future biomarker and genetic studies in Asians.

Sample Size and Power: Using the prevalence estimates from SiMES and previously published data in Chinese and Indians, we determined that a target sample size of 3,300 ethnic Indians and 3,300 ethnic Chinese residents would provide sufficient power not only to measure the prevalence of the major sight-threatening diseases with sufficient precision (e.g., measuring any condition with a prevalence of 1% with a 95% confidence interval 0.7 to 1.3%, and measuring the prevalence of glaucoma of 3% with a 95% confidence intervals of 2.5 to 3.5%), but also to assess inter-ethnic differences in major eye diseases.

Recruitment Strategies: Our recruitment officers sent letters of invitation, and conducted telephone calls and home visits to potential study participants. A person was considered “ineligible” to participate in the study if he/she had moved from the residing address, had not lived at the official address in the past six months, or was terminally ill (e.g., cancer) or deceased. A person was deemed ‘not contactable’ after at least six unsuccessful telephone calls and six home visits. Participants who declined the initial invitation were re-contacted at later times, with at least one contact every six months. Apart from individualized communication with participants, we provided free transportation and pick-up services to participants who requested this, and used the media (television, local newspaper, community road shows) to publicize the study and

increase participation.

Examination procedures: Each patient is examined according to a standardized protocol. Participants were initially greeted, the study procedures were explained and an informed, written consent was obtained. The examination procedures included measurements of height, weight, blood pressure and pulse rate, followed by a comprehensive ocular examination.

- Visual Acuity Assessment and Refraction Distance presenting visual acuity was measured using a logarithm of the minimum angle of resolution (Log MAR) number chart (Lighthouse International, New York, USA) at a distance of 4 meters, with the participant wearing their current optical correction (spectacles or contact lenses), if any. A number chart was used for participants who were unable to identify the Latin alphabets. If no number could be read at 4 meters, the participant was moved to 3, 2 or 1 meters consecutively and finally visual acuity was assessed as counting fingers, hand movements, perception of light, or no perception of light. Subjective refraction and distance best-corrected visual acuity in Log MAR scores were measured by trained and certified study optometrists. Near vision acuity test was done using the Log MAR near vision chart. Auto refraction, keratometry and ocular biometry were measured using an auto refractor (Canon RK-5 Auto Ref-Keratometer, Canon Inc. Ltd., Japan) and the IOL Master (Carl Zeiss; Meditec AG Jena, Germany) respectively.
- Lens and Retinal Imaging: We took lens photographs using a digital slit-lamp camera (Topcon model SL-D7; Topcon, Japan, with FD-21 flash attachment) and a retro illumination camera (Neitz, CS Cataract Screener). Fundus photography was performed using a digital non-mydriatic retinal camera (Canon CRDGi with a 20Diopter SLR backing, Canon, Japan) using Early Treatment for Diabetic Retinopathy Study (ETDRS) standard field 1 (centered on the optic disc) and ETDRS standard field 2 (centered on the fovea).
- Slit Lamp Examination: Anterior and posterior segment examinations were performed at the slit-lamp (Haag-Streit model BQ-900; Haag-Streit, Switzerland) using a 78 Diopter lens, which included measurements of vertical dimensions of the optic disc and cup with an eyepiece graticule, etched in 0.1 mm units. Intraocular pressure (IOP) was measured using the Goldmann applanation tonometer (Haag-Streit, Switzerland). Lens grading was based on the LOCS III system.29 Laboratory Tests
- Non-fasting venous blood sample was collected for biochemistry tests including serum lipids, glycosylated hemoglobin A1c (HbA1C), creatinine, random glucose as well as for deoxyribonucleic acid (DNA) archival. Extracted DNA samples were aliquoted and stored at –80 Celsius at the Singapore Tissue Network.

**The Asian AMD Phenotyping Study**

Study design and Specific Aims: The Asian AMD Phenotyping Study is a prospective observational clinical study of Asian patients with exudative maculopathy signs secondary to neovascular AMD or PCV in Singapore. The study followed the principles of the Declaration of Helsinki, and approval was obtained from the institutional review board. The study aims to recruit 500 patients who will provide a comprehensive set of data to phenotype the clinical condition. Data will also be compared with 1500 age-gender-race-matched population-based controls from the Singapore Epidemiology of Eye Disease (SEED) program, which includes three population-based studies: the Singapore Malay Eye Study, the Singapore Indian Eye Study and the Singapore Chinese Eye Study, and 500 age-gender-matched controls from shite persons in Australia (Blue Mountains Eye Study [BMES]). The specific aims of the Asian AMD Phenotyping Study are the following:

- To compare the demographics and risk factors of Asian AMD between the three main ethnic groups in Asia (Chinese, Malays and Indians) with age–race-matched controls and with white populations as represented by the BMES.
- To determine the clinical course, treatment pattern and treatment response in Asian persons over a 1-year period separately in eyes with exudative AMD or PCV.
- To determine the natural history and progression of fellow eyes over a 1-year period by detailed clinical and ancillary investigations.
- To determine the impact of exudative AMD and PCV on functioning and QoL using validated questionnaires.
- To collect and store blood specimens for future biomarker and genetic studies.

Sample Size Calculation: Sample sizes of 500 cases and 1500 controls are determined to be sufficient to detect risk factor associations with an odds ratio of 1.5 and higher. The prevalence of risk factors in controls are mostly between 10% and

30% based on the National Health Survey 2004 (NHS 2004),25 for example, smoking (12.6%), diabetes (8.2%) and hypertension (24.9%). Power Analysis and Sample Size (PASS, version 11.0.4, NCSS, Kaysville, Utah, USA) was used to calculate the study power needed.

Recruitment strategies: Cases are recruited from the retinal clinics of the three major public hospitals with tertiary eye care in Singapore (Singapore National Eye Center, Tan Tock Seng Hospital and National University Hospital). Consecutive cases presenting with untreated neovascular AMD or PCV are invited to participate in the study. A person was considered ‘ineligible’ if he or she had received treatment for the macular disease within 12 months or if he or she was unable to give informed consent or follow imaging requirements of the protocol. One thousand five hundred age-fender-race matched Asian controls were selected from population studies under the SEED program.

Examination procedures: Each patient is examined according to a standardized protocol. The design of the protocol is aimed to capture clinical, angiographic and functional data of the condition over a 12-month follow-up period. In addition, data regarding a comprehensive range of risk factors are collected. The extensive and standardized examination procedure was derived in part from the Singapore Epidemiology of eye diseases studies. After eligible participants are identified from the retinal clinic, the study procedures are explained and a written, informed consent is obtained. The examination procedures include measurements of height, weight, blood pressure and pulse rate, followed by a comprehensive ocular examination.

- *Visual acuity assessment and refraction:* distance presenting visual acuity is measured using either Snellen chart or a logarithm of the minimum angle of resolution (LogMAR) chart at a distance of 4 m, with the participant wearing his or her current optical correction, if any. A number chart is used for participants who are unable to identify the Latin alphabets. If no numbers are read at 4 m, the participant is moved to 3 m, 2 m or 1 m consecutively. If no numbers are identified on the chart, visual acuity is assessed as counting fingers, hand movements, perception of light or no perception of light. Auto refraction is performed using an auto-refractor (Canon RK-5 Auto Ref-Keratometer, Canon Inc. Ltd, Tokyo, Japan). Ocular biometry is measured using the IOL Master (Carl Zeiss; Meditec AG Jena, Germany). Subjective refraction and distance best corrected visual acuity in LogMAR scores are measured by trained and certified study optometrists at month 3 and month 12 visits.
- *Slit-lamp examination:* a structured slit-lamp examination (Haag-Streit model BQ-900; Haag-Streit, Koeniz, Switzerland) is performed by study ophthalmologists before and after pupil dilation. The first examination determines abnormalities in the anterior segment of the eye (e.g. pterygium, iris abnormalities). After pupil dilation, grading of cataract is performed using Lens Opacity Classification System III. Fundus examination is performed to diagnose AMD or PCV and to identify any abnormalities in the optic disc, macula and peripheral retina.
- *Fundus photography:* a digital mydriatic retinal camera (TRC-50X/IMAGEnet 2000, Topcon, Tokyo, Japan) is used to obtain color photographs of Early Treatment for Diabetic Retinopathy Study (ETDRS) standard field 2 (centered on the fovea) of each eye.28 Autofluorescence images are acquired using the same fundus camera and ETDRS standard field 2 with the Spaide filter (Topcon).
- *Angiography:* fundus angiography with fluorescein and indocyanine green is performed using a fundus camera (TRC-50X/IMAGEnet 2000, Topcon) or confocal scanning laser ophthalmoscope (SLO) (Heidelberg Retina Angiograph Spectralis; Heidelberg Engineering, Heidelberg, Germany). After injection of 5 mL of 10% sodium fluoride, early-phase Fluorescein Angiography (FA) images are taken until 1 min after injection. Late-phase FA images are acquired at least 5 min after injection. This is followed by injection of 25 mg indocyanine green (ICG) after which early-phase indocyanine green angiography (ICGA) images are taken up to 1 min, followed by mid-phase ICGA images up to 6 min. About 15 min after the ICG injection, the late-phase ICGA images are obtained.
- *Optical coherence tomography:* spectral domain optical coherence tomography (OCT) (Cirrus OCT; Carl Zeiss Meditec, Dublin, CA) is obtained at each visit using the macular cube setting.
- *Blood sample for biochemistry tests and deoxyribonucleic acid (DNA) archival:* non-fasting venous blood is collected to determine levels of serum glucose, lipids, creatinine and C-reactive protein (CRP). In addition, for the purpose of DNA archival, extracted DNA samples are aliquoted and stored at -80 Celsius at the Genomic Institute of Singapore.

Supplementary Table S1. **Comparison of Lipoprotein Profiles in neovascular AMD (nAMD) cases from population control to overall cohort.**

|  | Mean (standard deviation) | | | | | | | |  |  |
| --- | --- | --- | --- | --- | --- | --- | --- | --- | --- | --- |
|  | No AMD (N=289) | | | | Early AMD (N=184) | nAMD (N=193) | | | **nAMD from population study**  **(n=17)** |  |
|  | **Total particle concentration by subclass** | | | | | | | | | |
| ApoA1, mg/ld. | 155.8 (26.7) | | | 154.6 (23.6) | | | | 145.3 (45.5) | **NA** |  |
| HDL lipoprotein, µmoll/L | 35.3 (6.0) | | | 34.8 (5.2) | | | | 37.2 (7.4) | **33.5 (5.0)** |  |
| LDL lipoprotein, nmol/L | 1320.8 (439.6) | | | 1222.4 (360.8) | | | | 1236.5 (416.9) | **1172.5 (241.2)** |  |
| VLDL & Chylomicron lipoprotein, nmol/L | 73.2 (30.5) | | | 67.4 (28.4) | | | | 60.6 (23.2) | **59.0 (15.3)** |  |
|  | **Lipoprotein size distribution by subclass** | | | | | | | | | |
| HDL lipoproteins, µmol/L |  | | | | | | | | | |
| Large | 7.1 (3.8) | 6.9 (3.6) | | | | | 6.9 (3.5) | | **7.7 (3.0)** |  |
| Medium | 9.1 (5.0) | 9.2 (4.5) | | | | | 10.4 (5.0) | | **7.2 (3.5)** |  |
| Small | 19.1 (6.4) | 18.6 (5.7) | | | | | 19.9 (6.4) | | **18.7 (5.6)** |  |
| IDL lipoproteins, nmol/L |  | | | | | | | | | |
| IDL | 110.7 (79.6) | | 114.7 (82.6) | | | | 155.6 (110.6) | | **177.3 (90.8)** |  |
| LDL lipoproteins, nmol/L |  | | | | | | | | | |
| Large | 581.2 (264.5) | 554.0 (240.4) | | | | | | 448.3 (269.6) | **372.8 (256.0)** |  |
| Small | 628.8 (427.7) | 553.7 (343.1) | | | | | | 632.6 (375.5) | **622.5 (303.3** |  |
| VLDL & Chylomicron, nmol/L |  | | | | | | | | | |
| Large | 6.6 (6.3) | | 6.1 (5.9) | | | | 5.2 (3.8) | | **9.0 (6.0)** |  |
| Medium | 30.8 (19.4) | | 25.4 (16.2) | | | | 17.5 (12.1) | | **19.7 (10.9)** |  |
| Small | 35.8 (17.9) | | 36.0 (17.3) | | | | 37.9 (16.5) | | **30.4 (16.3)** |  |
|  | **Mean Particle Sizes** | | | | | | | | | |
| HDL particle size, nm | 9.2 (0.5) | | 9.3 (0.5) | | | | 9.3 (0.5) | | **9.5 (0.5)** |  |
| LDL particle size, nm | 20.9 (0.6) | | 21.0 (0.6) | | | | 20.8 (0.6) | | **20.8 (0.7)** |  |
| VLDL particle size, nm | 49.8 (7.5) | | 49.8 (8.2) | | | | 50.5 (7.6) | | **55.8 (10.4)** |  |
